# Supplementary material for: Causal Relationship between Aging and Anorexia Nervosa: A White-Matter-Microstructure-Mediated Mendelian Randomization Analysis
Source: Biomedicines. 2024 Aug 16;12(8):1874. doi: 10.3390/biomedicines12081874 (PMC11351342; doi:10.3390/biomedicines12081874)
Supplement: Supplementary file 1 [file biomedicines-12-01874-s001.zip › Supplementary Figure/Figure S4/Singe SNP plot of MR analysis for TL to ubm-b-1575.pdf]

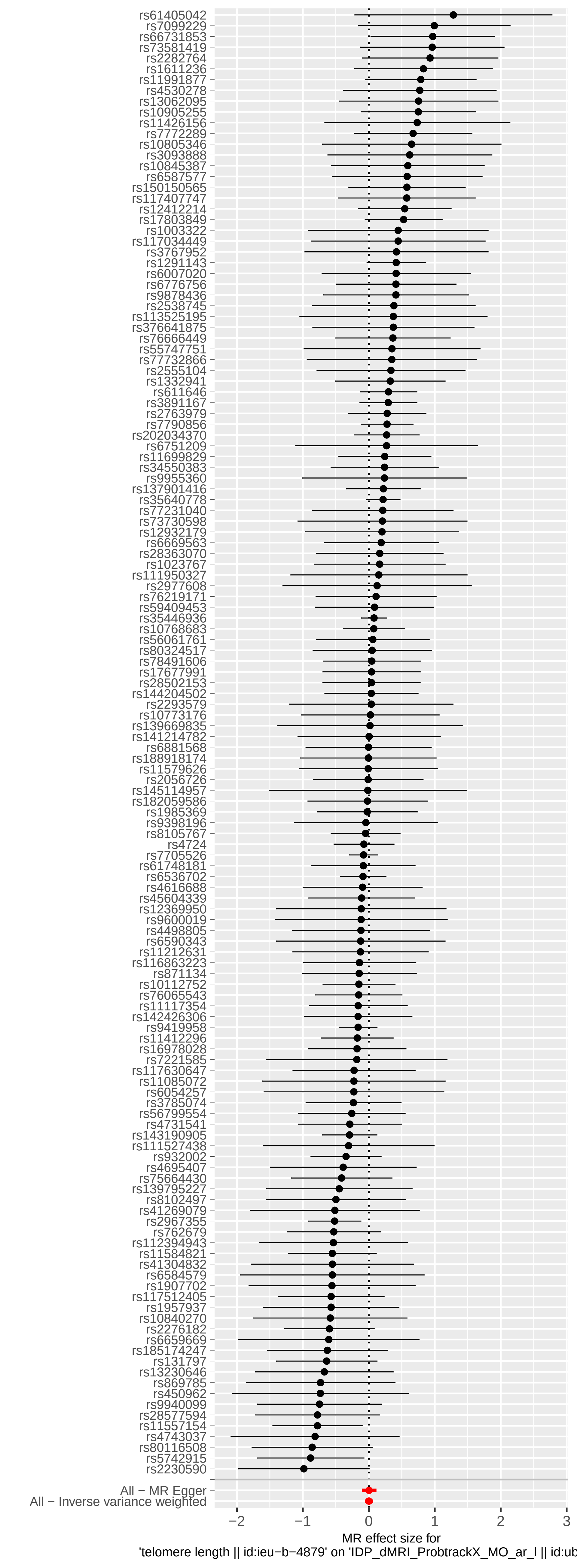
rs61405042
rs7099229
rs66731853
rs73581419
rs2282764
rs1611236
rs11991877
rs4530278
rs13062095
rs10905255
rs11426156
rs7772289
rs10805346
rs3093888
rs10845387
rs6587577
rs150150565
rs117407747
rs12412214
rs17803849
rs1003322
rs117034449
rs3767952
rs1291143
rs6007020
rs6776756
rs9878436
rs2538745
rs113525195
rs376641875
rs76666449
rs55747751
rs77732866
rs2555104
rs1332941
rs611646
rs3891167
rs2763979
rs7790856
rs202034370
rs6751209
rs11699829
rs34550383
rs9955360
rs137901416
rs35640778
rs77231040
rs73730598
rs12932179
rs6669563
rs28363070
rs1023767
rs111950327
rs2977608
rs76219171
rs59409453
rs35446936
rs10768683
rs56061761
rs80324517
rs78491606
rs17677991
rs28502153
rs144204502
rs2293579
rs10773176
rs139669835
rs141214782
rs6881568
rs188918174
rs11579626
rs2056726
rs145114957
rs182059586
rs1985369
rs9398196
rs8105767
rs4724
rs7705526
rs61748181
rs6536702
rs4616688
rs45604339
rs12369950
rs9600019
rs4498805
rs6590343
rs11212631
rs116863223
rs871134
rs10112752
rs76065543
rs11117354
rs142426306
rs9419958
rs11412296
rs16978028
rs7221585
rs117630647
rs11085072
rs6054257
rs3785074
rs56799554
rs4731541
rs143190905
rs111527438
rs932002
rs4695407
rs75664430
rs139795227
rs8102497
rs41269079
rs2967355
rs762679
rs112394943
rs11584821
rs41304832
rs6584579
rs1907702
rs117512405
rs1957937
rs10840270
rs2276182
rs6659669
rs185174247
rs131797
rs13230646
rs869785
rs450962
rs9940099
rs28577594
rs11557154
rs4743037
rs80116508
rs5742915
rs2230590
All − MR Egger
All − Inverse variance weighted
−2
−1
0
1
2
3
MR effect size for
'telomere length || id:ieu−b−4879' on 'IDP_dMRI_ProbtrackX_MO_ar_l || id:ub
